# The Korean Version of the Adolescent Dissociative Experience Scale: Psychometric Properties and the Connection to Trauma among Korean Adolescents

**DOI:** 10.4306/pi.2009.6.3.163

**Published:** 2009-07-15

**Authors:** Jung-Uk Shin, Sung Hoon Jeong, Un-Sun Chung

**Affiliations:** 1 Department of Psychiatry, School of Medicine, Kyungpook National University, Daegu, Korea.; 2 Department of Psychiatry, Kyungpook National University Hospital, Daegu, Korea.

**Keywords:** Adolescent Dissociative Experience Scale, Dissociation, Adolescents, Trauma, Validity

## Abstract

**Objective:**

The Adolescent Dissociative Experience Scale (A-DES) is a screening measure for dissociative experience in adolescents. The present study aimed to investigate the reliability, validity and psychometric properties of the Korean version of the Adolescent Dissociative Experience Scale.

**Methods:**

The Korean version of the A-DES was administered to a normative group of 371 adolescents aged 12 to 18 years and a traumatized group of 33 adolescents aged 12 to 18 years with known trauma.

**Results:**

The internal consistency was excellent (Cronbach's alpha=0.91) and the test-retest correlation of the A-DES was high (r=0.99). Correlation between the A-DES and other measures of dissociation was moderate (r=0.48). There were no significant age differences in mean total A-DES scores for the normative sample, or for boys or girls separately. Nor were there any significant gender differences for any age group. The mean total score of the A-DES was significantly higher in the traumatized group than in the normative group. There was a statistically significant difference between adolescents with self-reported trauma and those without a trauma history in the normative group.

**Conclusion:**

This study demonstrated that the Korean version of the A-DES is a reliable measure with excellent internal consistency and good stability over a 4-week test-retest interval with single factor structure. It can be used to screen for dissociative symptoms in Korean adolescents between the ages 12 and 18.

## Introduction

Dissociative disorder is defined as a disruption in the usually integrated functions of consciousness, memory, identity, and perception of the environment, and it includes dissociative amnesia, dissociative fugue, dissociative identity disorder, depersonalization disorder and dissociative disorder Not Otherwise Specified.^1^ The core feature of dissociation is a lack of integration of consciousness, and it includes a number of related phenomena-amnesia (the inability to recall a significant segment of time), absorption and imaginative involvement (the ability to be lost in the task at hand), derealization (a sense that your surroundings are unreal), and depersonalization (a sense of detachment from your body or self).^2^ Dissociation can be considered a natural protective response to overwhelming traumatic experiences like child sexual abuse and physical abuse and is associated with posttraumatic stress in many studies.^3^-^5^ However, it is also related to normal developmental processes and the frequency of dissociative experiences peaks during late latency years and declines between early adolescence and young adulthood.^6^,^7^

Most investigations of those who have experienced trauma during childhood focus on adults, and in such research the Dissociative Experiences Scale has been used for assessing dissociative symptomatology in adults.^8^ Given increasing interest in psychiatric problems of children and adolescents who have experienced physical and sexual abuse, a screening tool to measure dissociation in children and adolescents had been developed: a caregiver-report scale of the Child Dissociative Checklist by Putnam^6^,^9^ and a self-report questionnaire of the Adolescent Dissociative Experience Scale by Armstrong during the 1990s.^10^ Children and adolescents with dissociative symptoms are usually diagnosed with multiple comorbid disorder including reactive attachment disorder, depression, attention deficit-hyperactivity disorder, post-traumatic stress disorder, borderline personality disorder, and conduct disorder over repeated evaluations.^11^-^13^ Therefore, assessment for the presence of dissociation is essential in the psychiatric evaluations of all children and adolescents.^14^

As a screening tool for the dissociative symptoms and experiences of adolescents, there are the Adolescent Dissociative Experiences Scale (A-DES), the Dissociative Questionnaire (DisQ), and the adolescent version of the Multi-Dimensional Inventory of Dissociation (MID).^10^,^15^,^16^ Among these, the A-DES has been developed solely for adolescents and its reliability and validity has been well-established.^17^ The other two scales cover adolescents and adults together and the DisQ has only satisfactory reliability and validity (32). Therefore, the A-DES is the most widely used scale, and German, Dutch, Turkish, Finnish and Swedish versions of the A-DES have been developed.^2^,^7^,^11^,^18^,^19^ It is a 30-item self-reporting scale composed of 4 theoretical subscales: amnesia, absorption, depersonalization/derealization, and passive influence.^10^ However, results of factor analysis in different cultures, including British, Dutch, Finnish and Swedish samples, have not shown four-factor structure like in the original English scale, but instead have shown single factor structure.^2^,^7^,^19^,^20^

The original author of the A-DES found that A-DES scores were elevated in adolescents with self-reported trauma.^10^ Nilsson et al.^7^ confirmed that trauma is associated with a higher level of dissociative symptoms, showing that adolescents who had experienced trauma had significantly higher A-DES scores than adolescents in the normative group, and adolescents with self-reported trauma scored significantly higher than adolescents with no self-reported trauma.

In this study, our aim was to test the validity and reliability of the Korean version of the A-DES in Korean culture, and compare its psychometric properties with results in other countries. It was also of interest to study eventual gender and age differences, as the results were not consistent for adolescents in previous studies. In the British sample, there was no significant age or gender difference.^2^ In Turkey, A-DES scores were negatively correlated with age, but there was no gender difference.^11^ In Sweden, girls in the age range of 14 to 15 years scored higher than other age/gender groups.^7^

Proving concurrent validity of the A-DES requires investigating the correlation of the A-DES and the Youth Self Report (YSR). Kisiel et al.^21^ and Keck Seeley et al.^22^ found that there was a significant positive correlation between the A-DES and the scores on the Child Behavior Checklist (CBCL) in children and adolescents. Sim et al.^23^ reported that the newly formed posttraumatic stress disorder (PTSD)/dissociation subscale from the CBCL correlated significantly with the child self-reported dissociation on the Trauma Symptom Checklist For Children (TSCC).^24^ The CBCL and the adolescent form of the CBCL, the YSR, are widely used in Korea, but the TSCC is not yet validated in Korea.

For discriminant validity, we investigated whether the A-DES would differentiate between a normative group and a traumatized group, and between a normative group with self-reported trauma and with no self reported trauma, as in previous studies.

## Methods

### Participants

#### Normative Group

The intention was to cover 400 adolescents aged from 12 to 18 years old who were attending two middle and high schools in Deagu, a city in southeastern South Korea with approximately 2.4 million inhabitants. Permission was obtained in advance from the headmasters and teacher and parents' committee of the school board of the school in which the study was performed. The Ethics Committee of Kyungpook National University Hospital granted permission for the study. A total of 400 adolescents agreed to participate in the study and 377 completed the A-DES. The research data were collected by self-rating questionnaires that the students completed during class periods at school, with the instructions given by teachers who had been informed in advance. Participants with prior psychiatric diagnosis and treatment were excluded. The response rate was 98.4%, giving a sample of 371 adolescents. Six questionnaires were excluded because of missing data and the final sample included 371 subjects, 181 boys and 190 girls. The mean age in the normative group was 14.90 years old (SD=1.80); boys only, 14.93 (SD=1.78) and girls only, 14.86 (SD=1.83).

For the purpose of test-retest reliability, 25 students randomly selected filled out the A-DES a second time, 4 weeks later. A reasonable time interval regarding stability in response and minimizing individuals' memory effect is considered to be between 2 and 4 weeks.^25^ We investigated the demographic data such as parental education level in years, caretakers living with adolescents, and socioeconomic status by Hollingshead et al.^26^

#### Traumatized Group

Originally, we intended to investigate adolescents with a dissociative disorder. However, because there had not been a large enough number of such patients in their adolescence in our hospital, we explained the aim of our study to other child and adolescent psychiatrists in our city and searched for the number of patient with the dissociative disorder in their adolescence. Even with this trial, there had been few adolescents with dissociative disorder.

We decided to study traumatized adolescents instead, and we sent letters to directors of the protective facilities for sexually and/or physically abused adolescents located in Daegu and Gyeongbuk Province because adolescents in those facilities had been definitely abused and could be classified as a traumatized group. Adolescents with mental retardation and known psychosis were excluded. Sixty eight adolescents were asked to participate in the study, but 38 consented and only 33 of them provided complete data.

Finally, the Traumatized group consisted of 33 adolescents, 23 boys and 10 girls, between 12 and 19 years old. The mean age was 14.55 (SD=1.87); boys only, 14.34 (SD=1.72); and girls only, 15.03 (SD=2.18). Adolescents had been sent to the protective facilities by the police or social workers. Twenty-four (72.7%) were not living with their parents and 37.5% of their parents had divorced.

### Questionnaires

#### Adolescent Dissociative Experience Scale

The A-DES was originally validated with a clinical sample of 12 to 18 year olds^10^ and a nonclinical sample of 11 to 17 year olds.^2^ The A-DES is a 30 item self-report questionnaire with an 11-point scale ranging from 0 representing "never" to 10 representing "always." The total A-DES score is equal to the mean of all item scores. Four subscales are supposed to reflect the main constructs of dissociation: amnesia (2, 5, 8, 12, 15, 22, and 27), absorption and imaginative involvement (1, 7, 10, 18, 24, and 28), passive influence (4, 14, 16, 19, and 23), and depersonalization and derealization (3, 6, 9, 11,13, 17, 20, 21, 25, 26, 29, and 30). The researcher obtained permission for the development of a Korean version from the author of the original scale. The Korean version of the scale was translated by a fluent bilingual person, and a consensus procedure was performed with Korean child and adolescent psychiatrist. Another bilingual translator made a blinded back-translation to English and the final version of the A-DES was established after some adjustment. We focused on Korean expressions in good agreement with the original version while considering cultural and social differences.

#### Youth Self-Report

The YSR is a standardized selfrated questionnaire for 11- to 18-year-old adolescents to assess their own behaviors and emotional states with 112 items developed by Achenbach.^27^ This is the adolescent self-rated version of the CBCL which is completed by a parent to evaluate his or her own child. Raw YSR scores were transformed to T-scores. The Korean version of the YSR was developed and has been widely used as a screening tool for behavioral problems of adolescents.^28^ In a previous study, the following CBCL items were identified as good measures of either PTSD or dissociation or both syndromes if at least two thirds (67%) of experts rated the item as an indicator of either or both: PTSD (9, 29, 45, 47, 50, 76, and 100), dissociation (13, 17, and 80), PTSD/dissociation (8, 9, 13, 17, 29, 40, 45, 47, 50, 66, 76, 80, 84, 87, 92, and 100).^23^

#### Lifetime Incidence of Traumatic Events

Each participating student also filled out the student version of the Lifetime Incidence of Traumatic Events (LITE) checklist devised by Greenwald and Rubin,^29^ indicating which types of traumas or losses she or he had experienced, at what age, how many times, and how bad she or he felt at that time. Items included a car accident, house fire, death of a family member, exposure to threats, sexual assault, witness to violence, and many other potentially upsetting events.^29^ Dissociative symptoms have been found to correlate with traumatic histories of significant sexual abuse and/or physical abuse, as well as other types of trauma and natural disaster.^30^ For this reason, we used the LITE for the coverage of all kinds of trauma. Because the LITE is a screener, not an objectively scorable instrument, the students were rated on the LITE as follows: having no significant trauma or loss (a LITE score of 1), signifying the possibility of some significant trauma (a LITE rating of 2), signifying that significant trauma or loss was likely to have occurred (a LITE rating of 3) and signifying that significant trauma or loss definitely occurred (a LITE rating of 4) as the original authors had scored it.^29^ In this study, we translated the student version of the LITE into Korean to assess the experience of trauma by adolescents. Adolescents in the normal control group were classified into normative groups without significant trauma (a LITE rating of 1) and normative groups with significant trauma (a LITE rating of 4). The Korean version of the LITE had been developed following the same process as translation of the A-DES. All the adolescents in the Traumatized Group had the score of 4 when the LITE ratings were reviewed blindly.

#### Statistical analyses

The extent to which items on the A-DES are internally consistent/reliable was assessed using Cronbach's alpha. A standard of 0.90 or higher was considered excellent.^31^ For the test-retest reliability, a Pearson correlation was used. To establish validity, a factor analysis (normative group) was conducted (extraction method: principal component analysis; rotation method: varimax with Kaiser normalization).

Item-total correlation was calculated using a Pearson correlation. To test the significance of the differences between the normative group and the traumatized group (the mean total scores of the A-DES), a t test was used. To test the significance of the differences between normative groups with trauma, normative groups without significant trauma and the traumatized group, one-way analysis of variance (ANOVA) was used with post hoc comparison.

Descriptive statistics (means, standard deviations) were calculated for the A-DES total scores and the YSR scores for each group. A correlation matrix (Pearson r) was used to investigate a potential relationship between the A-DES scores and the YSR scores.

Descriptive statistics (means, standard deviations) were calculated for the mean total A-DES scores and the total scores of PTSD/dissociation items in the YSR for the 3 groups and a correlation matrix (Pearson r) was used to investigate a potential relationship between the A-DES scores and the total scores of PTSD/dissociation items in the YSR.

We divided the Normative Group into 3 age groups, 12 to 13 years (n=39, 33 boys, 6 girls), 14 to 15 years (n=158, 77 boys, 81 girls), and 16 to 18 years (n=174, 71 boys, 103 girls) because girls in the age range of 14 to 15 years scored higher than other age/gender groups in the Swedish study.^7^ Two way ANOVA with the mean total score as the dependent variable and gender as well as age as independent factor were used to estimate differences. The statistical program Statistical Package for Social Science (SPSS; SPSS Inc., Chicago, IL, USA) version 11.5 was used (p<0.05).

## Results

### Demographic data

An analysis of demographic data describing the two samples is given in Table 1. Age was not significantly different between the normative group and traumatized group and between males and females in both groups. The educational level of the father (t=5.01, p<0.001) and mother (t=6.58, p<0.001) and socioeconomic status of the traumatized group (χ^2^=40.72, p<0.001) were significantly lower than in the normative group. The family type of the two groups were significantly different (χ^2^=90.26, p<0.001). In the normative group, 86.5% of adolescents lived with both parents, but 72.7% of adolescents in the traumatized group were cared for by the social workers in the protective facilities.

### Reliability measures

The internal consistency for the A-DES in this study was measured by Cronbach's alpha (normative group) and found to be 0.91, indicating excellent internal reliability of the Korean version of the A-DES. In a 4-week interval, the test-retest reliability correlation of the Korean version of the A-DES was found to be r=0.99 (n=25, p<0.01).

### Validity measures

#### Factor Analysis

To test the construct validity of the A-DES, a factor analysis was conducted, and it yielded eight factors (eigen values ≥1) that explained 64.53% of the variance. This to-be-expected four-factor solution was examined and compared with the theoretically suggested subscales but could not be found in our eight-factor solution. The theoretically suggested subscale items were mixed in all the eight factors. With this result and from a scree plot, a single-factor solution that explained 64.53% of the total variance seemed more appropriate. Item-total correlations were calculated to establish partial construct validity of the scale. These coefficients ranged from 0.34 to 0.66, and all correlations reached a significance level of p<0.01 (Table 2).

#### Concurrent Validity

Interscale correlations were calculated. The results revealed a significant positive, although moderate, correlation between the total score of the A-DES and the PTSD/dissociation scale of the YSR (r=0.48, p<0.001). For boys (r=0.50, p<0.05) and for girls (r=0.48, p<0.05), moderate positive correlation also showed. For adolescents with self-reported significant trauma experiences, the intercorrelations between the mean total score on the A-DES and the PTSD/dissociation scale of the YSR were r=0.50 (p<0.01), and without self-reported experience of trauma, r=0.42 (p<0.01).

A significant positive, although moderate, correlation also was found between the mean total A-DES score and the YSR Total T score (r=0.47, p<0.01), the mean total A-DES score and the YSR Externalizing T score (r=0.43, p<0.01), and the mean total A-DES score and the YSR Internalizing T score (r=0.48, p<0.01).

### Differences within the normative group

#### Gender Difference

In the normative group, there was no significant difference between boys (n=181, 0.81±0.95) and girls (n=190, 0.68±0.77) in the mean total scores of the A-DES.

#### Age Differences

There was no significant correlation between the mean total A-DES score and age. There were no significant differences across the age groups (12-13, 14-15, 16-18 years old) in the mean total scores of the A-DES. There were no significant gender differences in mean total A-DES scores across the age groups (12-13, 14-15, 16-18 years old).

#### Differences in Adolescent Dissociative Experience Scale scores, Self-Reported Trauma versus no Self-Reported Trauma in the Normative Group

In the normative group, 76 adolescents (20.5%) were assessed as having had a significant trauma in their past based on the self-rating of the student form of LITE (a score of 4). Ninety four (25.3%) in the normative group reported that they had not experienced a significant trauma on the self-rating of the student form of LITE (a score of 1). The mean total of the A-DES scores for the group who had reported trauma was significantly higher (1.09±1.19) than for the group with no reported trauma (0.50±0.55)(t=-4.005, p<0.01). There were also significant mean differences on the total A-DES score between the no trauma group the normative group without self-reported trauma (0.50±0.55), normative group with self-reported traumagroup (1.09±1.19), and the traumatized group (2.05±1.92)(F=22.955, p<0.001)(Table 3).

#### Differences between the Normative Group and the Traumatized Group

The mean score for the traumatized group (n=33) was significantly higher (2.05±1.92) than in the normative group (n=371)(0.75±0.86). The difference between the groups was found to be significant (t=-3.85, p<0.01).

## Discussion

This initial validation study shows the Korean version of the A-DES to be a promising measure of adolescent dissociation experiences in Korean adolescents. Internal consistency as measured by Cronbach's alpha in our study was 0.91, which was compared to 0.93 in the original study by Armstrong in the United States^10^ and Muris et al. in the Netherlands,^32^ 0.94 in the English sample,^2^ 0.95 in the Swedish version^7^ and 0.93 in the Turkish version of the A-DES.^11^ The test-retest reliability correlation for the Korean Version of the A-DES also had been high: r=0.99 in a 4-week interval. The test-retest reliability coefficient in the Turkish version was 0.91 over a 2-week interval for 29 participants consisting of different diagnostic groups and normal controls,^11^ while Smith and Carlson^20^ reported a test-retest reliability coefficient of 0.77 in their sample of 60 high school students in a 2-week interval in the United States, and in the Swedish version, it was 0.71 in their samples of 83 pupils in a 3-week interval. In our study, the test-retest interval was the longest, but the test-retest reliability coefficient was the highest among the four reliability studies in different countries and different languages. Therefore, the Korean version of the A-DES is an internally consistent and reliable scale.

Five previous studies have provided information regarding the factor structure of the A-DES in normal samples. Although Armstrong had developed the A-DES with theoretically-derived 4 factor structure, a factor analysis of the A-DES scores in a British sample of 768 adolescents aged 11 to 16 years old showed one factor that explained 39.1% of the variance on the A-DES scores and single-factor structure was also the most suitable in the Netherlands sample of 331 adolescents from secondary school.^2^,^10^,^32^ Similarly, a one-factor solution that explained 44.1% of the total variance was found in the Swedish sample of 400 adolescents aged 12 to 19 years old and a single factor solution accounting for 42.21% of the variance had been considered optimal in Finnish adolescents, too.^7^,^19^ Thus, Keck Seeley et al.^22^ suggested that it appears that for normal adolescents the A-DES items did not cluster into the four domains reflecting different aspects of dissociation, but the A-DES assessed a single dimension of dissociation. Likewise, we could not find the hypothetical four subscales in the A-DES that had been described by Armstrong et al.,^10^ i.e., amnesia, absorption and imaginative involvement, passive influence, and depersonalization. In the Korean version of the A-DES, a single-factor solution that explained 64.53% of the total variance is the most appropriate, which is in line with previous studies. Based on these results, as Farrington et al.^2^ proposed, dissociation in adolescence may be a more homogenous experience than in adulthood, and multiple factors may be derived for the A-DES only among clinical samples of adolescents.

In the present study, item-total correlations ranged from 0.34 to 0.66 (Table 2) and this result is in line with the findings for the 810 British teenagers aged from 11 to 16 years, which ranged from 0.39 to 0.70.^2^

Concurrent validity was investigated by correlating the Korean version of the A-DES scores with scores on the YSR because no screening tool for adolescents with dissociation was available in Korean. A significant positive moderate correlation was found between the total score of the A-DES and the PTSD/dissociation scale of the YSR (r=0.48).

Kisiel et al.^21^ found that there was a significant positive correlation (r=0.27) between the A-DES and the scores on the CBCL in their sample of sexually abused children and adolescents. Keck Seeley et al.^22^ showed a significantly moderate correlation between the mean total A-DES score and the CBCL Total T score (r=0.41), the CBCL Externalizing T score (r=0.44) and the CBCL Internalizing T score (r=0.33).^22^ We also found that the Korean version of the A-DES Total mean score was moderately correlated with all three scales form the YSR (each, r=0.47, r=0.43, r=0.48) The reason for the slightly higher correlation in our study than the previous two studies might be self-rated YSR by adolescents compared to the CBCL scale by the parents/guardians.

Consistent with previous results on normal adolescents,^2^,^10^,^18^,^20^ there were no significant age or gender differences in the Korean version of the mean A-DES scores. However, the Turkish version of the A-DES scores decreased significantly with age, and in the Swedish version of the A-DES, scores were the highest among girls aged from 14 to 15 years old.^7^,^11^ In the Finnish sample, those aged 13-15 years had higher scores but they the researchers reported that they were significantly different but not clinically relevant.^7^,^19^ The lack of significant differences in dissociation scores across age ranges in our study might reflect that Korean adolescents could not procede to emotional regulation and to decreased normal dissociation as they grow older. According to the annual report on the health behavior among Korean adolescents by the Korean National Statistical Office in 2007, 46.5% of the middle and high school students in Korea answered that they were suffering from severe stress due to excessive pressure on academic performance and competition for entrance to university and one out of 20 of them had attempted suicide.^33^ Suicidal risk among adolescents is rising sharply in Korea and teenage suicide has become the 5th cause in 2003 from the 9th cause of death in 1993, and Kim^34^ reported in 2008 that 11.6% of 1,908 Korean adolescents aged 12-18 had attempted suicide. From these results, we hypothesized that normal emotional development might be impaired during adolescence in Korea and adolescence was not the key transitional period when dissociation peaks and begins to decrease.^2^,^10^ However, more research is needed on the adolescent's emotional regulation.

In our study, traumatized adolescents had significantly higher A-DES scores than adolescents in the normative group, as well as adolescents in the normative group with self-reported trauma. The normative adolescent group reporting traumatic experiences scored significantly higher than without self-rated trauma. As a result, increased scores were associated with a history of trauma in this study, which is in line with a previous study.^7^ In Finnish adolescents, those with a low level of dissociation more often had parents who were living together, which is in line with this study. They also found that daily smoking, frequent use of alcohol, abuse of legal drugs, no friends and infrequent social contacts, and poor school performance in mathematics were independently associated with being in the high dissociation group.^19^

The mean total scores of the A-DES in different languages in the normative group are shown in Table 4. In our sample, the mean scores were low (0.75), which is similar to the results in the Swedish version (0.84), the Finnish version (0.88) and Seeley et al. in U.S.A (0.66).^7^,^19^,^22^ The total mean scores of the Dutch (1.27) the Turkish (2.4) and Farraington's results (2.66) in the United Kingdom were higher than our results.^2^,^11^,^32^ Armstrong et al.^10^ found a total mean score of 2.24 (n=60) in another area of the U.S. Nilsson et al.^7^ assumed that adolescents from bigger cities are closer to community violence. However, our city, Daegu has 2,500,000 inhabitants and is a city of a similar size of Los Angeles (Armstrong et al.) and Hampshire (Farrington et al), although Istanbul in Turkey is a very large city of 12,000,000 inhabitants.^2^,^10^ These findings suggests that some underlying features of these different samples other than population explain the differences between them.^19^

In our study, the mean of the traumatized group (2.05) was also low. Another reason for the low mean score for both groups is due to the higher response of "0". The "0" (never) category was the most widely chosen response with a mean selection frequency of 75.8% in our study compared to 45.2% of the "0" never category in the study of Armstrong et al.^10^ It cannot be completely ruled out that the Korean adolescents have fewer dissociative symptoms than those in other countries. Another explanation might be a cultural difference between different countries. Previous studies investigating the prevalence of the psychiatric disorders in Korea showed very low prevalence. Cho et al.^35^ suggested that stigma for mentally ill patients was so severe in Korea that respondents had a tendency to conceal their symptoms. Taylor et al.^36^ explained that Korea was the one of the countries showing response bias such toward giving the most socially desirable answer. According to a study of Kim^37^ Korean adolescents perceived their self-health risk likelihood and rated the chances of most health risks happening to them as significantly lower than the Austrian group. Considering the fact that this study was performed with students preparing to proceed to the university and teacher's opinions significantly influence academic reports in Korea, adolescents might have more a socially desirable response tendency at school even with anonymity. In addition, only 55.8% adolescents in the traumatized group had consented to participate in this study and there had been a selection bias between responders and non-responders. Although we had no available information about non-responders, responders might have more socially desirable tendencies than non-responders. Previous studies also found that survey non-respondents tend to have significantly higher rates and severities of mental illnesses than respondents.^38^

In Korea, the prevalence of patients with dissociative disorder has not yet been studied. Moreover, adolescents are rarely diagnosed with a dissociative disorder at all. We hypothesized that the mean total A-DES score was associated with trauma, and we showed the mean A-DES score of the traumatized adolescents and adolescents with reported trauma was significantly higher than without trauma.

However, further studies are needed to establish the discriminant validity of this scale for adolescents with dissociative disorders. A relatively small number in the traumatized group is a limitation of our study. Another weakness of this study is that the teacher took care of the procedure on behalf of the researchers, and that might increase the tendency toward socially desirable responses in the classroom. More research is needed to examine the factor structure of the scale and to establish the optimal cutoff for identifying adolescents with significant dissociative symptoms in Korea. This self-rating A-DES scale enables clinicians to screen the dissociative symptoms of adolescents which otherwise may easily go unnoticed. With a diagnostic interview for dissociative disorders after screening, treatment strategies towards reducing dissociation can be highly effective in treating adolescent victims of trauma and maltreatment like sexual abuse and physical abuse who engaged in disruptive and self-destructive behavior later.^30^

Because there is no Korean screening tool for evaluating dissociative symptomatology in adolescents, this will facilitate studies about adolescents with trauma and earlier recognition and earlier treatment of the dissociative disorder of adolescents in Korea.

This study demonstrates that the Korean version of the A-DES is a reliable measure with excellent internal consistency and good stability over a 4-week test-retest interval with a single factor structure. It can be used to screen for dissociative symptoms in Korean adolescents between the ages 12 and 18.

## Figures and Tables

**TABLE 1:**
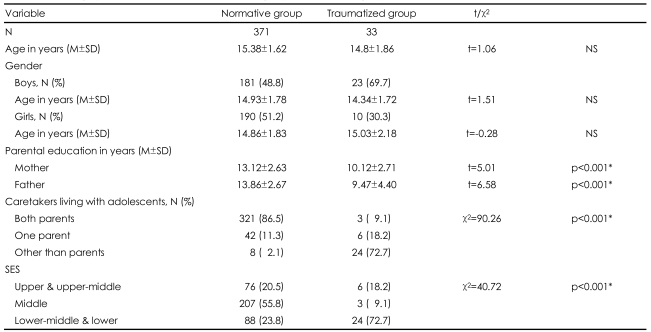
Demographic data of traumatized adolescent group and normative group

^*^p<0.001. N: number, M: mean, SD: standard deviation, SES: socioeconomic status. SES by Hollingshead and Redlich was used

**TABLE 2:**
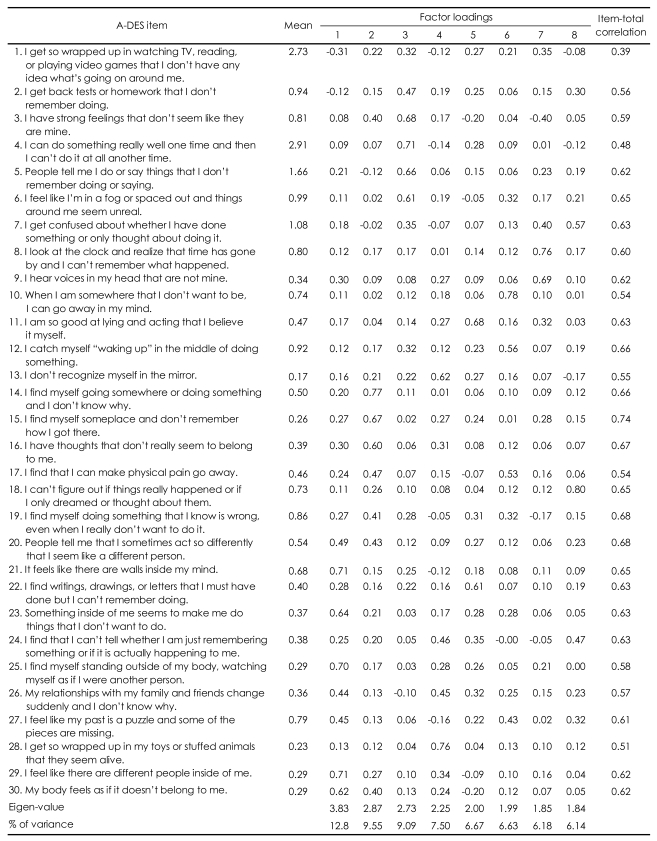
Mean scores, factor loadings, and item-total correlations for each A-DES item

A-DES: Adolescent Dissociative Experience Scale

**TABLE 3:**
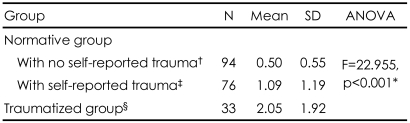
Differences in mean A-DES total score between the normative group with no self-reported trauma, with self-reported trauma, and the traumatized group

^*^All post comparison tests were statistically significant at the level of p<0.001, ^†^^‡^^§^One way analysis of variance (ANOVA) was used, ^†^^‡^^§^Were results of post comparison analysis by the Bonferroni method. A-DES: the Adolescent Dissociative Experiences Scale, N: number, SD: standard deviation

**TABLE 4:**
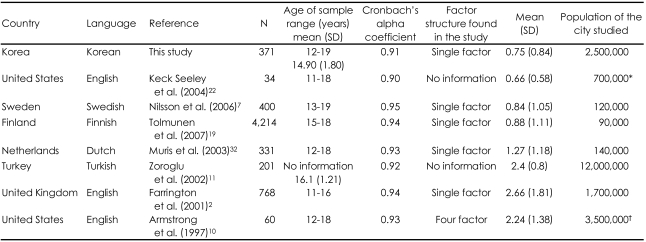
Internal reliability coefficients, factor structure, mean total scores of the A-DES and population of the cities studied for different language translations

^*^Akron, Ohio, USA, ^†^Los Angeles, California, U.S.A. A-DES: the Adolescent Dissociative Experiences Scale, N: number, SD: standard deviation
